# DNA-PKcs: A Multi-Faceted Player in DNA Damage Response

**DOI:** 10.3389/fgene.2020.607428

**Published:** 2020-12-23

**Authors:** Xiaoqiao Yue, Chenjun Bai, Dafei Xie, Teng Ma, Ping-Kun Zhou

**Affiliations:** ^1^School of Public Health, University of South China, Hengyang, China; ^2^Department of Radiation Biology, Beijing Key Laboratory for Radiobiology, Beijing Institute of Radiation Medicine, Beijing, China; ^3^Department of Cellular and Molecular Biology, Beijing Chest Hospital, Capital Medical University/Beijing Tuberculosis and Thoracic Tumor Research Institute, Beijing, China

**Keywords:** DNA-PKcs, DNA damage response, DNA repair, genomic instability, radiosensitization

## Abstract

DNA-dependent protein kinase catalytic subunit (DNA-PKcs) is a member of the phosphatidylinositol 3-kinase related kinase family, which can phosphorylate more than 700 substrates. As the core enzyme, DNA-PKcs forms the active DNA-PK holoenzyme with the Ku80/Ku70 heterodimer to play crucial roles in cellular DNA damage response (DDR). Once DNA double strand breaks (DSBs) occur in the cells, DNA-PKcs is promptly recruited into damage sites and activated. DNA-PKcs is auto-phosphorylated and phosphorylated by Ataxia-Telangiectasia Mutated at multiple sites, and phosphorylates other targets, participating in a series of DDR and repair processes, which determine the cells’ fates: DSBs NHEJ repair and pathway choice, replication stress response, cell cycle checkpoints, telomeres length maintenance, senescence, autophagy, etc. Due to the special and multi-faceted roles of DNA-PKcs in the cellular responses to DNA damage, it is important to precisely regulate the formation and dynamic of its functional complex and activities for guarding genomic stability. On the other hand, targeting DNA-PKcs has been considered as a promising strategy of exploring novel radiosensitizers and killing agents of cancer cells. Combining DNA-PKcs inhibitors with radiotherapy can effectively enhance the efficacy of radiotherapy, offering more possibilities for cancer therapy.

## Introduction

Eukaryotic cells are constantly encountering various endogenous (e.g., DNA replication errors) and exogenous (ionizing radiation, chemical carcinogens, and UV) stresses of genomic DNA damage (Jeggo et al., 2016). DNA damage includes base damage, pyrimidine dimer formation, single or double strand breaks, etc. Among them, DNA double strand break (DSB) is the most serious and dangerous threat that affects the stability of the genome and cell fate (Jackson and Bartek, 2009; Huang and Zhou, 2020). Failure to make timely and precise repairs can lead to accumulation of residual DNA damage, mutations, rearrangements, and/or loss of chromosomes, which can lead to a series of cellular consequences such as cell death, senescence, transformation, mutagenesis, or carcinogenesis (Her and Bunting, 2018). Fortunately, cells have evolved several precise DNA damage response (DDR) and repair machineries to deal with various types of DNA damages. DDRs include DNA damage sensing, initiating DNA damage signaling cascades, remodeling and relaxing chromatin around DSBs, recruiting DNA repair proteins to the damaged site, activating cell cycle checkpoints, and repairing DSB (Jackson and Bartek, 2009; Huang and Zhou, 2020). DNA repair defects are closely related to a series of human diseases and aging (Vijg and Suh, 2013; Longerich et al., 2014; Madabhushi et al., 2014; Shimizu et al., 2014; Chow and Herrup, 2015; Konstantinopoulos et al., 2015; Fang et al., 2016; Uryga et al., 2016; Chatzidoukaki et al., 2020). The DSBs repair pathways of eukaryotic cells mainly include the non-homologous end joining (NHEJ), homologous recombination (HR) and alternative end-joining (Lieber, 2008; Chiruvella et al., 2013; Chang et al., 2017; Shibata and Jeggo, 2020).

DNA-dependent protein kinase (DNA-PK) is an important player in the NHEJ pathway and was also found to function in multiple nodes of DDRs. DNA-activated/or DNA-dependent protein kinase was first reported in 1985 by Walker et al. (1985). They discovered this protein by chance, finding that addition of double-stranded DNA (dsDNA) into the cell extracts increased the phosphorylation of certain proteins. In 1990, Carter et al. (1990) and Lees-Miller et al. (1990) identified the DNA-PK catalytic subunit (DNA-PKcs) with hsp90 or casein as phosphorylation substrate bait. DNA-PKcs is an abundant protein with 50,000–100,000 molecules per cell in humans (Anderson and Lees-Miller, 1992; Blunt et al., 1995; Miller et al., 1995; van der Burg et al., 2009; Woodbine et al., 2013; Chang and Lieber, 2016). *PRKDC* gene mutations in patients or expression of kinase-dead DNA-PKcs protein in mice causes severe combined immunodeficiency (SCID). DNA-PKcs, Ataxia-Telangiectasia Mutated (ATM), and ATM and RAD3 related (ATR) belong to the family of the phosphatidylinositol 3-kinase related kinase (PIKK), which plays significant roles in DNA damage repair (Blackford and Jackson, 2017; Kantidze et al., 2018). They all harbor similar domain compositions, including a N-terminal HEAT-repeat rich segment followed by the conserved FRAP-ATM-TRRAP (FAT) domain, the kinase domain, the PIKK regulatory domain (PRD) and the FAT C-terminal motif (FATC; Blackford and Jackson, 2017). DNA-PKcs, ATM, and ATR preferentially phosphorylate the S/T-Q motif (serine or threonine residue followed by a glutamine) (Kim et al., 1999). DNA-PKcs and ATM mainly mediate the repair of DNA double-strand breaks through NHEJ and HR, respectively, while ATR responds to the stalled DNA replication forks and DNA single-strand breaks (Falck et al., 2005; Menolfi and Zha, 2020). Whereas, a series of reports indicated that DNA-PKcs is also required for optimal replication stress response (Lin et al., 2014, 2018; Wang et al., 2015; Ying et al., 2016; Kantidze et al., 2018; Joshi et al., 2019; Villafanez et al., 2019).

This review mainly summarized and discussed the updated accomplishments about DNA-PKcs research, including post-translation modifications and activity regulation of DNA-PKcs and its involvements in DDRs. Furthermore, considering the special role of DNA-PKcs in the DDR, we have also reviewed the progress on exploration of DNA-PKcs inhibitors as radiosensitizers for cancer radiotherapy.

### Post-translational Modifications and Activation of DNA-PKcs Triggered by DNA Damage Signaling

DNA-dependent protein kinase catalytic subunit belongs to the PIKKs family, and is a type of DNA-activated serine/threonine protein kinase with a molecular weight of approximately 469 kD encoded by the *PRKDC* or *XRCC*7 gene (Hartley et al., 1995; Sipley et al., 1995; Lees-Miller, 1996). It forms a holoenzyme DNA-dependent kinase (DNA-PK) with the heterodimer regulatory subunits of Ku70 and Ku80(referred to as Ku together) (Jin and Weaver, 1997; Singleton et al., 1999). Ku70 is encoded by *XRCC6* gene and Ku80 is encoded by *XRCC5.* Ku70 and Ku80 have a strong affinity for DNA ends, and they also provide docking sites for other proteins during DDR (Featherstone and Jackson, 1999).

**FIGURE 1:**
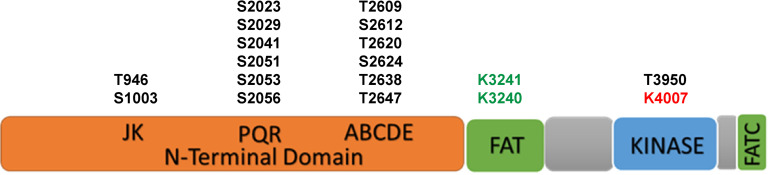
The scheme of functional domains and post-translational modifications of DNA-PKcs. The black font represents phosphorylation sites, green font represents acetylation sites and red font represents the neddylation site.

The DNA-PKcs structure consists of an N-terminal region, a circular cradle unit, and a head unit with the kinase domain between FAT (FRAP, ATM, and TRRAP) and FATC domains (Sibanda et al., 2010, 2017; Sharif et al., 2017; Yin et al., 2017). Both the N-terminal region and the cradle unit contain HEAT (Huntingtin, Elongation factor 3, regulatory subunit A of PP2A, and TOR1) repeats. Both the ABCDE and PQR phosphorylation clusters fall into the cradle unit (Figure 1; Spagnolo et al., 2006; Sibanda et al., 2010; Morris et al., 2011).

The activity and function of DNA-PKcs in end-ligation or NHEJ aretightly regulated by phosphorylation modification. The N-terminal domain has many phosphorylation sites, DNA-PKcs can be auto-phosphorylated or phosphorylated by ATM and ATR (Chen et al., 2007; Dobbs et al., 2010). The FAT and FATC domains surround the catalytic domain, stabilize the conformational changes of the catalytic center and regulate kinase activity (Rivera-Calzada et al., 2005; Jiang et al., 2006; Spagnolo et al., 2006). As shown in Figure 1, the phosphorylation sites of ABCDE clusters are T2609, S2612, T2620, S2624, T2638, and T2647, and the phosphorylation sites of PQR clusters are S2023, S2029, S2041, S2051, S2053, and S2056 (Douglas et al., 2002). Among them, S2056 and T2609 are two prominent autophosphorylation sites of DNA-PKcs, both are crucial for its activity in DNA repair (Ding et al., 2003; Block et al., 2004; Nagasawa et al., 2011, 2017). The current model suggests that DNA-PKcs S2056 phosphorylation causes conformational changes, thereby promoting DNA-PK disassembly from the DSB site, allowing DNA end ligation (Jiang et al., 2015), while the ABCDE cluster phosphorylation is required for DNA end resection (Shibata et al., 2011). Another autophosphorylation site, T3950, is located in the kinase domain and its phosphorylation was suggested to shut down the activity of DNA-PKcs kinase (Douglas et al., 2007).

In addition to phosphorylation modification, there are also other forms of post-translational modifications on DNA-PKcs (Table 1). Proteomics studies have revealed that DNA-PKcs are widely acetylated (Mori et al., 2016) and ubiquitinated (Ho et al., 2014). Lysine acetylation is an important form of post-translational modifications and is also the most widely studied post-translational modification on histones. Proteomics showed that at least 16 lysine residues were acetylated in the DNA-PKcs. Mori et al. (2016) selected eight of them for further research and have confirmed that K3241 and K3260 were acetylated on DNA-PKcs. In addition, our research group also found that both PARylation (Han et al., 2019) and neddylation (Guo et al., 2020) can regulate DNA-PK activity, a neddylation site was identified at K4007 within the kinase domain. PARP1-dependent DNA-PKcs PARylation can be induced by DNA damage signals and affects DNA-PKcs Ser2056 phosphorylation in cells (Han et al., 2019). DNA-PKcs neddylation occurs in the kinase domain and is catalyzed by HUWE1 ligase (Guo et al., 2020). In 2014, the ring finger protein 144A (RNF144A) was discovered as the first E3 ubiquitin ligase of cytoplasmic DNA-PKcs. Ho et al. (2014) RNF144A is induced in a p53-dependent manner during DNA damage and targets cytoplasmic DNA-PKcs for ubiquitination and degradation.

**TABLE 1:** The modification sites of DNA-PKcs.

Modification sites	Modification type	Function	References
**T2609**	phosphorylation	Activates Artemis-mediated endonuclease activity; Promoting cNHEJ repair through end processing; DSB repair and cellular sensitivity to gamma radiation; Facilitates telomere leading strand maturation	Liu et al., 2012; Lin et al., 2014; Longerich et al., 2014; Lin et al., 2018
**S2056**	phosphorylation	DSB repair and cellular sensitivity to gamma radiation; Promotes end ligation in cNHEJ; Increases expression of RBX1 in G1 stage	Ma et al., 2002; Goodarzi et al., 2006; Lu et al., 2007; Reichert et al., 2011; Lin et al., 2014; Madabhushi et al., 2014; Jiang et al., 2019
**T2647**	phosphorylation	Activates Artemis-mediated endonuclease activity	Goodarzi et al., 2006; Lin et al., 2018
**T3950**	phosphorylation	Shuts down the activity of DNA-PKcs kinase	Konstantinopoulos et al., 2015
**K3241 K3260**	acetylation	Maintains genome stability and radiation resistance	Kotsantis et al., 2018
**K4007**	neddylation	Promotes autophosphorylation of DNA-PKcs at Ser2056	Guo et al., 2020

Considering the close relationship between DNA-PKcs and DSB, it is particularly important to determine the mechanism controlling DNA-PKcs activation. In the cellular response to DSB, Ku heterodimer recognizes and localizes DNA damage sites, and promptly recruits DNA-PKcs. Once DNA-PKcs is recruited, it is activated through phosphorylation in a DNA-dependent manner. The C-terminal 178 amino acid residues of Ku80 is required for Ku80/DNA-PKcs interaction and indispensable for DNA-PKcs activation (Singleton et al., 1999). DNA-PKcs pushes Ku protein inwards onto the DNA, and then phosphorylates the other components nearby, including its own phosphorylation. DNA-PKcs autophosphorylation can be regulated by N-terminal conformational changes of protein (Meek et al., 2012). In addition to the automatic regulation of DNA-PKcs, many other factors are also suggested to be key regulators of DNA-PKcs activity. For example, epidermal growth factor (EGFR) can bind DNA-PKcs and enhance the activity of DNA-PKcs to deal with the damage (Liccardi et al., 2011; Javvadi et al., 2012). The non-kinase regulator protein phosphatase 6 (PP6) and protein phosphatase 1(PP1) is recruited to the DSB site and promotes DNA-PKcs activity through direct interaction and dephosphorylation action (Douglas et al., 2010; Zhu et al., 2017). Thus, the activation of DNA-PKcs is not only the result of binding to the broken DNA ends, but also a complex process affected by many factors. The multiple mechanistic ways of activation suggest that there are many unknown functions waiting to be explored in DNA-PKcs in addition to participating in double-strand break repair.

### Regulation of DNA Damage Response by DNA-PKcs

Current knowledge displays that DNA-PKcs can function in multiple pathways of cellular DDRs to maintain the genome stability and cell survival.

### DNA-PKcs in Non-homologous End Joining

**FIGURE 2:**
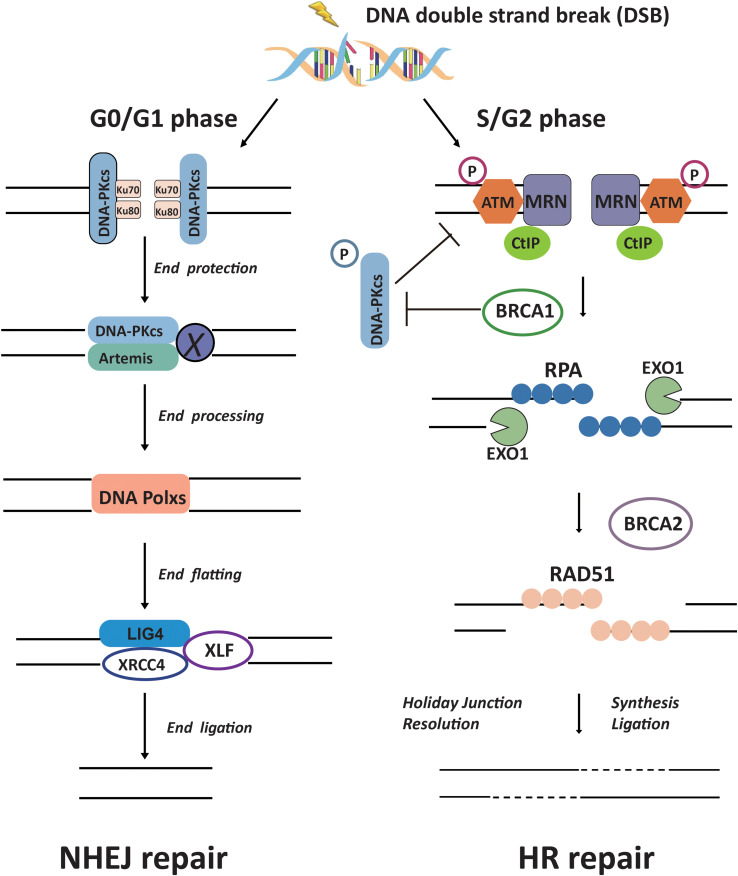
The canonical DSB repair pathways of NHEJ and HR.

There are two major repair pathways for DSB: NHEJ and HR (Figure 2). NHEJ repair is an error-prone repair pathway and theoretically executes its function throughout the cell cycle, but it is most important during G1 when no homologous template for recombination is available. HR performs under the guidance of an intact homologous template DNA, and it is known as an error-free repair pathway. HR is most active in the S/G2 phase and nearly absent in G1 phase (Mao et al., 2008; Menon and Povirk, 2016). As a main DSB repair pathway in mammals, NHEJ is initiated by the circular Ku70/Ku80 heterodimer binding to the broken DNA ends. Ku has a high abundance and strong affinity for free DNA. Within a second or less, Ku proteins can associate with any DSB that occurs in the nuclei genomic DNA. The interaction of Ku and broken DNA recruits DNA-PKcs to form DNA-PK complex (Gomes et al., 2017), then a set of NHEJ downstream factors including Artemis, XRCC4, DNA polymerase X family, and DNA ligase IV to join the broken DNA ends. DNA-PKcs is the only active protein kinase described in the NHEJ pathway, it is autophosphorylated in the presence of DNA termini, Mg^2+^ and ATP. DNA-PKcs forms a tight complex with Artemis to stimulate the nuclease activity of the latter (Kamoi and Mochizuki, 2010; Chang and Lieber, 2016). In this process, DNA-PKcs phosphorylates the C-terminal inhibitory region of Artemis, facilitating the dissociation of the inhibitory region from the N-terminal catalytic domain (Ma et al., 2002; Pannunzio et al., 2018). Ionizing radiation-induced DNA DSB broken ends are usually described as “dirty ends” and incompatibility for ends direct ligation caused by chemical modifications and 5′ or 3′ mismatching overhangs. The activated Artemis processes these “dirty ends” for subsequent ligation. DNA-PKcs kinase activity becomes necessary for direct end-ligation of NHEJ in the presence of DNA-PK protein. The DNA-PKcs^KD/KD^ (kinase-dead mutant) cells show severe end-ligation defects (Jiang et al., 2015).

Usually, NHEJ takes the first action to respond to the DSB (Shibata et al., 2011). If NHEJ cannot be completed, then DSB “cuts,” where one strand of the DNA duplex is degraded to produce a single-stranded DNA overhang suitable for alternative repair pathways, HR occurs (Sallmyr and Tomkinson, 2018). Experiments have shown that NHEJ is much faster than HR and occurs within 30 min (while HR requires 7 h or more), accounting for approximately 75% of repair events (Zhang and Matlashewski, 2019). NHEJ repair does not use sequence homology, regardless of the position or sequence, the DNA break ends are brought together, so this type of repair is prone to errors.

### Regulation of DNA-PKcs on the Pathway Choice of DSBs Repair

Homologous recombination repair is initiated by DSB end resection. MRE11 and CtIP together with EXO1 fulfill the DNA end resection process (Syed and Tainer, 2018). It is usually acknowledged that the interaction between 53BP1 and BRCA1 is an important factor to control the end resection (Daley and Sung, 2014; Zimmermann and de Lange, 2014; Mirman and de Lange, 2020). BRCA1 antagonizes certain functions of 53BP1 and promotes HR by reducing 53BP1-mediated NHEJ. After end resection, HR uses the undamaged homologous DNA as a template, and recombinase RAD51 invades the homologous DNA strand, resulting in precise repair (Sun et al., 2020).

**FIGURE 3:**
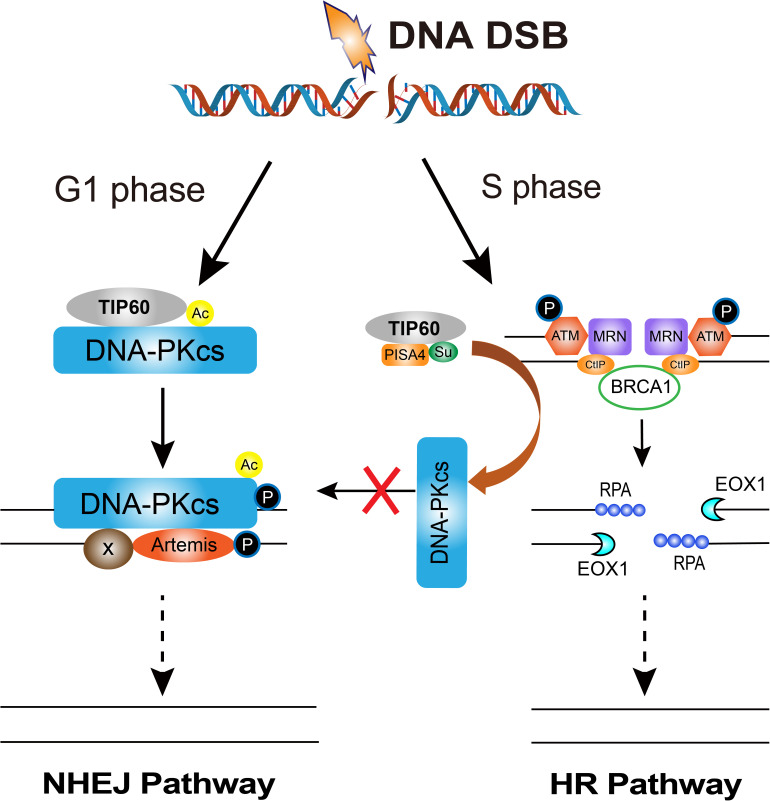
The regulation of DNA-PKcs-interacting TIP60 on the cell cycle-dependent choice of DNA DSBs repair pathway. Upon DNA DSBs signaling, TIP60 interacts with and acetylates DNA-PKcs to activate DNA-PKcs through autophosphorylation at S2056 for executing NHEJ in G1 phase. Whereas, in S phase cells, SUMO2 modification (SU) of TIP60 at K430 is mediated by PISA4 E3 ligase, which hinders the interaction between TIP60 and DNA-PKcs, consequently inactivating DNA-PKcs to give way to HR proteins and facilitates HR repair.

How cells select the pathway to execute DSBs repair between HR and NHEJ is a critical issue for efficient and precise repair of DSBs, whereas its mechanism is not fully understood yet (Ceccaldi et al., 2016; Scully et al., 2019). The initiation of DNA end resection is a definite factor that enables the cells to perform HR repair to prevent NHEJ repair (Scully et al., 2019). There are many factors that affect the initiation of end resection, but DNA-PKcs phosphorylation status is a clear factor that impacts cells to choose NHEJ or HR (Neal et al., 2011). A series of reports indicated that DNA-PKcs can regulate the choice of DSBs repair pathways at multiple biochemical nodes in association with the cell cycle. In the G_2_ phase of cell cycle, autophosphorylation of DNA-PKcs promotes DNA-PKcs dissociation from the DSBs sites, and facilitates the recruitment of end resection enzymes such as EXO1 (Shibata et al., 2011; Sallmyr and Tomkinson, 2018). DNA-PKcs also promotes end-resection from *in vitro* analyses (Deshpande et al., 2020). The DNA-PKcs mutant that makes autophosphorylation defective at the ABCDE cluster (DNA-PKcs ABCDE 6A) binds DSBs but precludes the completion of NHEJ, significantly reducing DSB end resection at all DSBs (Shibata et al., 2011). Whereas in developing lymphocytes, robust end-resection is detected in both DNA-PKcs kinase-dead or the phosphorylation (ABCDE) mutant (Crowe et al., 2018, 2020). ATM kinase activity can compensate for DNA-PKcs autophosphorylation when DNA-PKcs activity is inhibited and promote resection. The Mre11-Rad50-Nbs1 (MRN) complex further stimulates resection in the presence of Ku and DNA-PKcs by recruiting EXO1 and enhancing DNA-PKcs autophosphorylation, and it also inhibits DNA ligase IV/XRCC4-mediated end rejoining (Zhou and Paull, 2013). In the S phase of cell cycle, BRCA1 interacts with DNA-PKcs and directly blocks DNA-PKcs autophosphorylation, thus priming DNA end resection for HR and HR factors loading on DSBs (Davis et al., 2014; Figure 2). The interaction of TIP60 histone acetyltransferase and DNA-PKcs prompts the autophosphorylation and activation of DNA-PKcs (Jiang et al., 2006). We recently revealed that an increased SUMO2 modification of TIP60 K430 mediated by PISA4 E3 ligase hinders its interaction with DNA-PKcs in S phase cells, leading to impediment of DNA-PKcs S2056 autophosphorylation and preferentially employing the HR pathway for DSBs repair in S phase (Figure 3). TIP60 K430R mutation can recover the interaction of DNA-PKcs and TIP60, resulting in abnormally increased phosphorylation of DNA-PKcs S2056 in S phase and dramatical inhibition of HR efficiency (Gao et al., 2020).

Meanwhile, HR activity in G1 is restricted. Zhou et al. (2017) found that DNA-PKcs phosphorylates ATM directly, inhibiting ATM activity and ATM signaling upon DNA damage, providing a possible mechanism of HR restriction in G1 phase. Moreover, our recent report demonstrated that EXO1 protein is strictly suppressed in G1 phase. Increased expression of RBX1 protein prompts the neddylation and activity of cullin1, a key component of the Skp1-Cullin1-F-box (SCF) ubiquitin 3 ligase, and consequently mediates the ubiquitination degradation of EXO1 in G1 phase. Increased DNA-PKcs activity is responsible for increased RBX1 protein expression, and limiting the formation of DSB-end ssDNA by EXO1 and suppressing the HR repair pathway in G1 cells(Figure 3; Xie et al., 2020).

### DNA-PKcs in DNA Replication Stress

In addition to the role in DSB repair, DNA-PKcs is also involved in DNA replication stress response. Cells are particularly susceptible to DNA damage during the process of DNA replication. Almost all forms of DNA damage can disturb the DNA replication and cause replication stress (Bass et al., 2012).

Replication stress is defined as any DNA replication barrier that hinders, prevents or terminates DNA synthesis, and can activate the replication stress response to resolve the damage (Ubhi and Brown, 2019). Replicating stress checkpoint (S-phase checkpoint) involves the gradual activation of damage sensors, mediators and effectors (Allen et al., 2011). Replication protein A (RPA) binds to single-stranded DNA (ssDNA), recruiting numerous sensor proteins including ataxia telangiectasia and Rad3-related (ATR)-interacting protein (ATRIP), the 9-1-1 DNA clamp complex (RAD9-RAD1-HUS1), topoisomerase II binding protein 1 (TOPBP1) and Ewing tumor-associated antigen 1 (ETAA1), etc., triggers ataxia telangiectasia and Rad3-related (ATR) and phosphorylation of RPA32 (a subunit of RPA), leading to Chk1 activation (Zeman and Cimprich, 2014; Kotsantis et al., 2018).

DNA-dependent protein kinase catalytic subunit is necessary for ATR-Chk1 signal transduction. Upon replication stress, the DNA-PKcs is phosphorylated by ATR at the stalled replication forks, leading to transcriptional activation of Claspin expression and Chk1-Claspin complex stability, which is required for the optimal activation of intra S-phase checkpoint (Lin et al., 2014). The apoptosis mediator p53-induced protein with a death domain (PIDD) mediates DNA-PKcs recruitment at the stalled replication forks, and promotes the ATR signaling pathway in the cellular response to replication pressure and the cellular resistance to replication pressure (Lin et al., 2018). Phosphorylation of RPA32 Ser4/Ser8 is a key early step to fully activate ATR in response to replication stress and subsequent replication checkpoint stagnation. DNA-PK is the main kinase that targets Ser4/Ser8 in replication stress. The PIKK phosphorylation of RPA32 plays a key role in replication checkpoint activation, and DNA-PK was considered as an important contributor to this response (Liu et al., 2012). In addition, it was reported that ATR was critical in the early S phase, especially in cells under high replication stress; however, ATR can be circumvented by DNA-PKcs and Chk1 under moderate replication stress. They concluded distinct but concerted roles of ATR, DNA-PK, and Chk1 in countering replication stress (Buisson et al., 2015).

### DNA-PKcs in Autophagy

Autophagy is a physiological degradation mechanism of cells through which autophagic vesicles can deliver unfolded proteins and damaged organelles to lysosomes to eliminate them (Leidal et al., 2018). Autophagy plays an important role in different DDR pathways (Hewitt and Korolchuk, 2017). Inhibition of DNA-PKcs by bromovanin, a vanillin derivative, exhibited a potent antiproliferation effect through induction of both apoptosis and autophagy in HepG2 cells (Yan et al., 2007). Daido et al. (2005) reported that relative low-dose IR induced massive autophagic cell death in M059J cells that lack DNA-PKcs. The treatment of M059K cells with DNA-PKcs antisense oligonucleotides caused radiation-induced autophagy and radiosensitized the cells. Similar observations were also obtained by Puustinen et al. (2020) study. Inactivation or depression of DNA-PKcs increases ionizing radiation-induced cell autophagy as well as occurrence of cell apoptosis. It was found that DNA-PKcs interacts with the AMP-dependent protein kinase (AMPK) complex and phosphorylates nucleotide-sensing γ1 subunit (protein kinase AMP-activated non-catalytic subunit gamma) PRKAG1/AMPKγ1 at Ser192 and Thr284, thereby facilitating the AMPK complex activation by STK11 at lysosomal and autophagy.

### The Association of DNA-PKcs With Telomeres

Telomeres are DNA–protein complexes located at the ends of chromosomes in eukaryotic cells. The major function of telomeres is to maintain the stability and integrity of chromosomes (Roake and Artandi, 2020). The telomere sequence has unique characteristics, making telomeres susceptible to various DNA damages induced by the external environment and genotoxins (Lazzerini-Denchi and Sfeir, 2016). Any abnormality in telomere function may lead to cell aging/senescence and canceration. Telomeres can sense both intrinsic and extrinsic stresses and its dysfunction drives cell senescence (Victorelli and Passos, 2017). DNA-PKcs plays a role in telomere maintenance and is necessary for telomere capping (Goytisolo et al., 2001; Zhang et al., 2016; Sui et al., 2020). Sui et al. (2015) reported that hTR-mediated DNA-PKcs stimulation and subsequent hnRNP A1 phosphorylation affect the cell cycle-dependent distribution of telomeric repeat-containing RNA (TERRA) on telomeres by promoting the removal of TERRA in telomeres, which is important for the S-phase process, thereby promoting efficient telomere replication and capping (Ting et al., 2009). In cells lacking DNA-PKcs, uncapped telomeres are inappropriately detected and processed as DSB, and therefore not only participate in spontaneous telomere-telomere fusion, but also participate in ionizing radiation induction Telomere-DSB fusion event. The association between accelerated telomere shortening and decreased expression of DDR genes, including *DNA-PKcs*, *Mre11*, *Xrcc4*, etc, was found in the accelerated thymic aging of a rat model of developmental programming (Tarry-Adkins et al., 2019), suggesting DNA-PKcs may affect aging by maintaining telomeres integrity.

### DNA-PKcs Functions in Cell Cycle Checkpoints

When DDR occurs, the PIKK kinases ATM, ATR and DNA-PKcs coordinately play the role of maintaining genome integrity (Blackford and Jackson, 2017). These kinases control the repair of the broken DNA ends and transmit the damage signal through the tumor suppressor p53, CHK1/CHK2 and so on to induce cell cycle arrest, apoptosis, or aging. In this process, DNA-PKcs works by affecting G_2_/M DNA damage checkpoint in ATM deficient cells (Lee et al., 1997; Arlander et al., 2008; Tomimatsu et al., 2009; Shang et al., 2010). In ATM knockdown human mammary epithelial cells, either DNA-PK inhibitor treatment or RNAi knockdown of DNA-PKcs significantly attenuated G2 checkpoint (Arlander et al., 2008). In ATM-deficient AT5BIVA cells, DNA-PKcs inhibition led to a prolonged G_2_/M arrest (Shang et al., 2010). This phenotype could also be an indirect effect of no repair due to DNA-PKcs inhibitory role in ATM phosphorylation in DDR (Zhou et al., 2017). Inactivation of DNA-PKcs strikingly attenuated the ionizing radiation-induced phosphorylation of Chk1 or Chk2/T68 in ATM-deficient cells.

Ataxia-Telangiectasia Mutated, ATR, and DNA-PKcs can directly phosphorylate p53. ATM and ATR phosphorylate p53 through checkpoint kinases 2 (CHK2) and checkpoint kinases 1 (CHK1), respectively (Roos and Kaina, 2006). p53 stability is mainly regulated by oncoprotein MDM2, a key negative regulator of p53 (Haupt et al., 1997; Kubbutat et al., 1997). Under non-stress conditions, MDM2 and p53 N-terminal domain form a stable complex. MDM2-mediated proteasome degradation keeps p53 at a low level.

Under DNA damage stresses, p53 and MDM2 dissociate and p53 accumulates in the nucleus. p53 functions as a transcription factor to promote the expression of genes involved in DNA repair, cell cycle arrest, apoptosis, and aging to maintain genome integrity. Achanta et al. (2001) found that the DNA-PKcs/Ku/p53 complex may act as a sensor for DNA strand breaks caused by the incorporation of drug molecules, and then transduce signals to trigger apoptosis (Wang et al., 2000). Boehme et al. (2008) found that the activation of DNA-PKcs led to the phosphorylation and activation of Akt/PKB, which subsequently led to the inactivation of GSK-3β. As a result, MDM2 is phosphorylated and p53 accumulation increases. In this regard, DNA-PKcs modulates the p53-dependent apoptosis after DNA damage. At the same time, there are two negative feedback loops between p53 and MDM2 and wild-type p53-induced phosphatase 1 (Wip1) to regulate the level of p53 protein (Lu et al., 2007). Wip1 de-phosphorylates MDM2 and downregulates p53 protein levels by stabilizing MDM2, facilitating its access to p53.

### Targeting DNA-PKcs to Reprogram the Cellular Radiosensitivity

In the past century, radiation therapy has become the main treatment for cancer (Baskar et al., 2012). Statistically, about 50–70% of malignant tumor patients in the world are receiving radiotherapy (Huang and Zhou, 2020). However, the existence of radioresistance of cancer cells greatly hampers the efficacy and application of tumor radiotherapy (Kim et al., 2015). Radiation resistance is conducive to normal cells to escape the damage caused by ionizing radiation, but it is not conducive to the radiotherapy of malignant tumors.

The ability of cancer cells to repair DNA damage is a crucial factor that determines their sensitivity to radiation therapy or chemotherapy (Zhu et al., 2009). Inhibiting the repair ability of cancer cells offers a strategy to reduce the radiation resistance of cells and improve the efficacy of radiotherapy.

DNA-dependent protein kinase has become an attractive therapeutic target in various cancer treatments, especially when used in combination with genotoxic chemotherapy or ionizing radiation (Huang and Zhou, 2020; Medová et al., 2020). Studies have shown that targeting DNA-PKcs with various inhibitors can effectively enhance radiotherapy, and many small molecule inhibitors of DNA-PKcs have been developed and shown to be effective radiosensitizers *in vitro* (Table 2). One of the earliest identified inhibitors was wortmannin, which was isolated in 1957. It was defined as an effective non-competitive PI3K irreversible inhibitor in 1993, but it also targets other members of the PIKK family, including DNA-PKcs, ATM, ATR (Ui et al., 1995). The DNA-PK inhibitor NU7026 was reported to potentiate topo II poisons in treatment of leukemia through inhibition of NHEJ and G_2_/M checkpoint arrest (Willmore et al., 2004). In preclinical evaluation of DNA-PK inhibitor NU7441, it showed sufficient chemosensitization and radiosensitization with either etoposide, doxorubicin or ionizing radiation (Zhao et al., 2006). A highly selective DNA-PK inhibitor, AZD7648 showed efficient sensitization on radiation or doxorubicin in xenograft and patient-derived xenograft (PDX) models (Fok et al., 2019). M3814 inhibits DNA-PK catalytic activity and sensitizes various cancer cell lines to ionizing radiation (IR) and DSB inducers (Zenke et al., 2020). In addition, LY3023414 and CC-115 are both mTOR inhibitors, but they also have inhibitory effects on DNA-PKcs and other PI3KK (Smith et al., 2016; Thijssen et al., 2016; Bendell et al., 2018). It can be envisioned that the inhibition of DNA-PK shows considerable prospects in anti-tumor resistance. However, DNA-PK inhibitors are usually limited by poor pharmacokinetics: these compounds have poor solubility and unstable metabolism in the body, resulting in short serum half-life. Development of new compounds with better performance is a key step in obtaining effective anti-cancer drugs.

**TABLE 2:** The commonly used DNA-PK inhibitors.

Inhibitor	Target	Cell line or animal model	IC50(DNA-PK)	Clinical Trial	References
**NU7026**	DNA-PK, PI3K	K562, ML1 FeBALB/C mice, HeLa	0.23 μM		Nutley et al., 2005; Zimmermann and de Lange, 2014; Zhang et al., 2016
**NU7441 (KU-57788)**	DNA-PK, PI3K, mTOR	SW620, LoVo, V3-YAC, V3 cells, Ferude mice bearing SW620 xenografts	14 nM		Zhang and Matlashewski, 2019
**LY3023414**	PI3K, mTOR, DNA-PK	Athymic nude mice, CD-1 nude mice and NMRI athymic nude mice	4.24 nM	Phase 2	[Bibr B139][Bibr B140]
**AZD-7648**	DNA-PK	A549, H1299,BT-474, DLD1, FaDu, HCC70, HCC1806, HCC1937, HT-29, JEKO-1, MDA-MB-231, MDA-MB-436, MDA-MB-468, SKOV3, SUM149PT, TOV21G, UWB1.289	0.6 nM	Phase 1/2a	Zhao et al., 2006
**M3814**	DNA-PK	HCT116, FaDu, NCI-H460, A549, Capan-1, BxPC3	< 3 nM	Phase 1/2	Zhou and Paull, 2013
**CC-115**	mTOR, DNA-PK	Chronic lymphocytic leukemia (CLL) cells	13 nM	Phase 2	Zhu et al., 2009

## Future Perspectives

Over the decades, DNA-PKcs has been revealed to play multi-faceted roles and has been proved to be an essential regulator in the processes of DDR, however, there are still many unresolved questions. How does the interaction of DNA-PKcs and Ku-DNA complex leads to activation of DNA-PK protein kinase activity? There are a large number of sites and forms of post-translation modifications (PTMs) on DNA-PKcs, how these PTMs are inter-related and regulated, and how they influence DNA-PKcs functions. Are there any other DNA-PK substrates yet to be discovered? It is worth to draw the panorama of function-related DNA-PKcs interaction networks. The use of DNA-PK inhibitors for cancer therapy will not only inhibit the activity of DNA-PK in cancer cells, but also inhibit the activity of DNA-PK in normal cells. How to solve this problem? Ongoing exploration of DNA-PKcs’ multi-facet roles in DDR and besides will greatly contribute to our overall understanding of cancer and the discovery of new therapies.

## Author Contributions

P-KZ conceived and designed this study. XY drafted the initial manuscript. CB and DX reviewed and commented the manuscript. TM and P-KZ critically revised and finalized the manuscript. All authors contributed to the article and approved the submitted version.

## Conflict of Interest

The authors declare that the research was conducted in the absence of any commercial or financial relationships that could be construed as a potential conflict of interest.
